# Spending by the Veterans Affairs Health Care System for Medicare Advantage Enrollees

**DOI:** 10.1001/jamahealthforum.2025.5653

**Published:** 2025-12-19

**Authors:** Amal N. Trivedi, Lan Jiang, David J. Meyers, Aaron L. Schwartz, Kenneth W. Kizer, Jean Yoon

**Affiliations:** 1 Transformative Health Systems Research to Improve Veteran Equity and Independence (THRIVE) Center of Innovation (COIN), VA Providence Health Care System, Providence, Rhode Island; 2 Department of Health Services, Policy and Practice, Brown University, Providence, Rhode Island; 3 Crescenz VA Medical Center, Philadelphia, Pennsylvania; 4 Department of Medical Ethics and Health Policy, University of Pennsylvania Perelman School of Medicine, Philadelphia; 5 Division of General Internal Medicine, Department of Medicine, University of Pennsylvania Perelman School of Medicine, Philadelphia; 6 Clinical Excellence Research Center, Stanford University School of Medicine, Stanford, California; 7 VA Health Economics Research Center, VA Palo Alto Health Care System, Menlo Park, California; 8 Department of Medicine, School of Medicine, University of California at San Francisco

## Abstract

This cohort study estimates US Department of Veterans Affairs (VA) spending for veterans dually enrolled in the VA health care system and Medicare Advantage from 2019 to 2023.

## Introduction

Veterans dually enrolled in the Veterans Affairs (VA) health care system and Medicare Advantage (MA) plans constitute a growing share of both the VA and MA populations.^1,2^ MA plans can benefit from enrolling veterans who use little MA-financed care because MA plans receive full capitated payments from Medicare while the separately funded VA provides much of the veterans’ care. Federal law prohibits the VA from billing Medicare, raising concerns about duplicative spending for dually enrolled veterans.^1^ These concerns have garnered attention amid a 2025 bipartisan legislative proposal to authorize the VA to bill MA plans for services provided to enrolled veterans.^3^ We estimated that the VA provided over $78 billion in care to MA-enrolled veterans from 2011 to 2020.^2^ To inform current legislative deliberations, we provide updated estimates of VA spending for MA-enrolled veterans from 2019 to 2023.^3^

## Methods

In this cohort study, we identified all veterans concurrently enrolled in the VA and MA who received VA care from 2019 to 2023 (eMethods in Supplement 1). Spending estimates for care delivered directly by the VA used Managerial Cost Accounting data. Spending for VA-purchased community care, which finances health services for veterans receiving care in private-sector settings outside the VA, used actual reimbursements. All spending was inflation-adjusted to 2023 dollars. To contextualize spending, we calculated spending as a share of the VA’s annual congressional appropriation for health care. Pending legislation would authorize the VA to recover costs for non–service-connected conditions.^3^ Because the 1996 Eligibility Reform Act reduced the salience of distinguishing between service-connected and non–service-connected care, our estimates reflect total VA spending, although we also report results for veterans without service-connected conditions.

The Providence VA Medical Center Institutional Review Board approved this study with a waiver of informed consent owing to the use of deidentified data. We adhered to the STROBE reporting guideline.

## Results

We identified 1 781 207 veterans (mean [SD] age, 73.0 [9.5] years; 4.4% female, 95.6% male) who received VA care while enrolled in a MA plan from 2019 to 2023 (Table). This number increased from 1 002 564 in 2019 to 1 287 400 in 2023. Over this period, the VA spent $86.9 billion for this population, with annual spending increasing from $12.8 billion in 2019 to $22.7 billion in 2023, a 77% increase (Figure, A). Outpatient care accounted for the largest share of spending, while community care experienced the largest growth (221%). In 2023, the 600 195 MA enrollees without any service-connected disabilities accounted for $8.9 billion in VA spending.

**Table.** Characteristics of Veterans Dually Enrolled in the Veterans Affairs Health Care System and Medicare Advantage (N = 1 781 207)

Characteristic	Values
Age, mean (SD), y	73.0 (9.5)
Sex, No. (%)	
Female	78 875 (4.4)
Male	1 702 332 (95.6)
Race and ethnicity, No. (%)^a^	
Black	288 625 (16.2)
Hispanic	120 549 (6.8)
White	1 340 025 (75.2)
Other^b^	29 931 (1.7)
Unknown	2077 (0.1)

^a^ Race and ethnicity data were collected to characterize the study population given prior evidence that Medicare Advantage enrollment and outcomes differ by race and ethnicity.

^b^ Categories not further specified.

**Figure.**
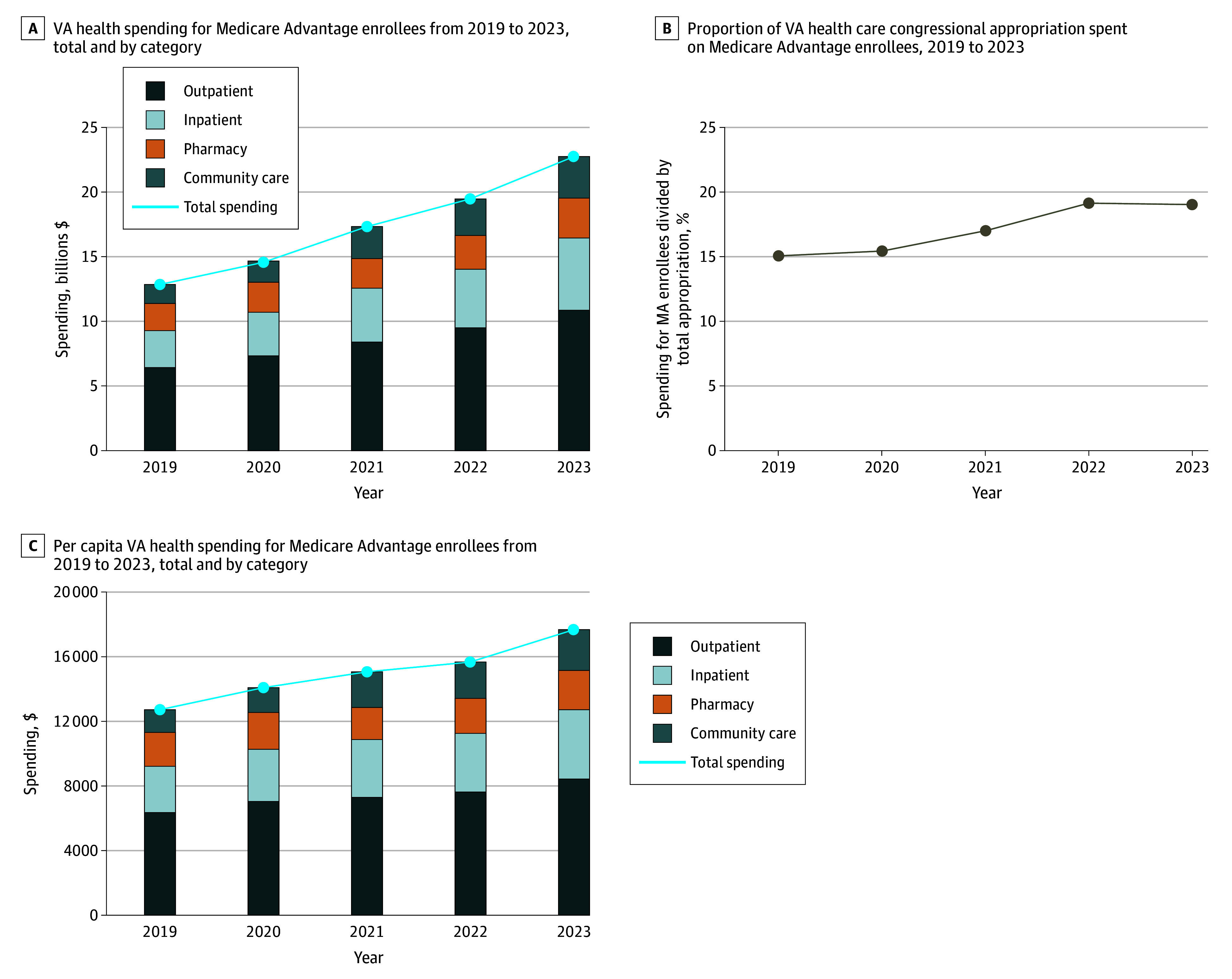
Veterans Affairs (VA) Health Spending for Medicare Advantage Enrollees, 2019 to 2023 A, Total spending reflects the amount the VA spent on enrollees who were dually enrolled in Medicare Advantage (MA). The inpatient, outpatient, and pharmacy bars reflect spending on services provided directly by VA clinicians and hospitals. The community care bar reflects spending on services delivered by non–Veterans Health Administration (VHA) clinicians but paid for by the VA. These totals include both direct VA care and care purchased from community hospitals and physicians. Spending estimates in 2019 and 2020 differ from those reported in Meyers et al^2^ primarily because of an 18% increase in the Consumer Price Index that occurred between 2020 and 2023 and, to a lesser extent, the use of managerial cost accounting instead of Health Economics Research Centre costing methods. B, Proportions reflect the total spending by the VHA for MA enrollees in each year divided by the total appropriation to the VHA in that year. C, Per capita spending reflects the amount the VA spent on enrollees who were dually enrolled in MA and used any VA services. The inpatient, outpatient, and pharmacy bars reflect spending on services provided directly by VA clinicians and hospitals. The community care bar reflects spending on services delivered by non-VHA clinicians but paid for by the VA. These totals include both direct VA care and care purchased from community hospitals and physicians.

Spending for MA-enrolled veterans accounted for 15.1% of the VA’s appropriation in 2019, rising to 19.0% in 2023 (Figure, B). Per capita annual VA spending for MA enrollees increased by 38% from $12 768 in 2019 to $17 665 in 2023 (Figure, C).

## Discussion

From 2019 to 2023, the VA spent $86.9 billion on care for veterans enrolled in MA plans, with annual spending rising 77% driven to nearly one-fifth of the VA’s health care appropriation. Although MA plans receive full capitated payments for these veterans, they are not required to reimburse the VA for services provided, effectively shifting their financial risk to a separately funded federal health system. This dynamic is especially notable in community care, where the VA directly finances services delivered in private-sector settings even as MA plans receive federal payments intended to cover the same care. These trends suggest that dual enrollment of veterans in MA functions as an implicit public subsidy to MA insurers. Furthermore, our findings raise important policy questions as veteran-targeted MA plans^4^ expand and spending for VA-purchased community care accelerates.

A study limitation is that we could not directly quantify potential overpayments due to the absence of MA risk scores, nor could we estimate all costs for non–service-connected conditions. Regardless, these estimates are relevant to legislative efforts to authorize VA reimbursement from MA plans and underscore the need for coordinated, efficient, and equitable financing of veterans’ health care.

## Notes

Supplement 1.
eMethods

eReference

Supplement 2.
Data Sharing Statement
