# Supplementary material for: Spending by the Veterans Affairs Health Care System for Medicare Advantage Enrollees
Source: JAMA Health Forum. 2025 Dec 19;6(12):e255653. doi: 10.1001/jamahealthforum.2025.5653 (PMC12717610; doi:10.1001/jamahealthforum.2025.5653)
Supplement: Supplement 1. — eMethods eReference [file jamahealthforum-e255653-s001.pdf]

Supplemental Online Content

Trivedi AN, Jiang L, Meyers DJ, Schwartz AL, Kizer KW, Yoon J. Spending by the Veterans Affairs health care system for Medicare Advantage enrollees. *JAMA Health Forum*. 2025;6(12):e255653. doi:10.1001/jamahealthforum.2025.5653

eMethods

eReference

This supplemental material has been provided by the authors to give readers additional information about their work.

© 2025 Trivedi A et al. *JAMA Health Forum*.

eMethods

Study Population

We used VA enrollment and utilization data linked to the Medicare Master Beneficiary Summary File to identify all Veterans who had at least one month of Medicare Advantage enrollment during the study period of 2019 to 2023. Medicare data were obtained through the VA Information Research Center (VIReC). In each calendar year, we identified the number of Veterans with utilization in any of the four main spending categories (VA outpatient, VA hospital care, prescription medication, community care) while concurrently enrolled in an MA plan.

Estimating VA spending

VA costs were obtained from the VA Managerial Cost Accounting (MCA) System data. The MCA System generates the cost of VA inpatient and outpatient care through a method in which relative values are assigned to health care encounters. The method then assesses the resources involved in producing encounters to estimate the production costs of care.[1] These costs include direct, indirect, and central administrative costs to operate the VA health care system nationally. The correlation coefficient for total annual costs per patient using MCA data and Health Economic Resource Center (HERC) Average Cost data has been estimated at 0.85.[1]

Community care costs were measured from payments to community providers in the Consolidated Data Set files. Prescription medication costs were derived from VA pharmacy claims and reflect VA's actual spending under negotiated prices with manufacturers.

Calculation of Total and Per-capita Spending

For the population of dual VA-MA enrollees who used VA services, we determined all costs that were incurred by the VA during the same month in which a veteran was concurrently enrolled in an MA plan. The measures of per-capita spending reflect the total annual VA spending for Veterans concurrently enrolled in an MA plan divided by the number of VA users who were enrolled in an MA plan during the same year. We did not annualize costs for those with less than 12 months of MA enrollment during the calendar year.

The Eligibility Reform Act and Service-connected Care

The Eligibility Reform Act eliminated inpatient and outpatient care differences/distinctions related to having or not having a service-connected condition. Once a veteran was enrolled in the system, the law erased the historical constraints on what care could be provided based on having a service-connected condition, although there still might be different copayments. The Act made inpatient and outpatient care broadly accessible to all enrollees, irrespective of having a service-connected condition.

eReference

1. Chapko MK, Liu CF, Perkins M, Li YF, Fortney JC, Maciejewski ML. Equivalence of two healthcare costing methods: bottom-up and top-down. *Health economics*. 2009;18(10):1188-1201.

© 2025 Trivedi A et al. *JAMA Health Forum*.
