# Supplementary material for: Spending by the Veterans Affairs Health Care System for Medicare Advantage Enrollees
Source: JAMA Health Forum. 2025 Dec 19;6(12):e255653. doi: 10.1001/jamahealthforum.2025.5653 (PMC12717610; doi:10.1001/jamahealthforum.2025.5653)
Supplement: Supplement 2. — Data Sharing Statement [file jamahealthforum-e255653-s002.pdf]

Data Sharing Statement

Trivedi. Spending by the Veterans Affairs Health Care System for Medicare Advantage Enrollees. *JAMA Health Forum*. Published December 19, 2025.
doi:10.1001/jamahealthforum.2025.5653

Data
Data available: No

Additional Information
Explanation for why data not available: The terms of our Data Use Agreement prohibit sharing veterans' data outside the VA.
